# Structural Studies of the Tandem Tudor Domains of Fragile X Mental Retardation Related Proteins FXR1 and FXR2

**DOI:** 10.1371/journal.pone.0013559

**Published:** 2010-11-02

**Authors:** Melanie A. Adams-Cioaba, Yahong Guo, ChuanBing Bian, Maria F. Amaya, Robert Lam, Gregory A. Wasney, Masoud Vedadi, Chao Xu, Jinrong Min

**Affiliations:** 1 Structural Genomics Consortium, University of Toronto, Toronto, Ontario, Canada; 2 Department of Physiology, University of Toronto, Toronto, Ontario, Canada; University of Cambridge, United Kingdom

## Abstract

**Background:**

Expansion of the CGG trinucleotide repeat in the 5′-untranslated region of the FMR1, fragile X mental retardation 1, gene results in suppression of protein expression for this gene and is the underlying cause of Fragile X syndrome. In unaffected individuals, the FMRP protein, together with two additional paralogues (Fragile X Mental Retardation Syndrome-related Protein 1 and 2), associates with mRNA to form a ribonucleoprotein complex in the nucleus that is transported to dendrites and spines of neuronal cells. It is thought that the fragile X family of proteins contributes to the regulation of protein synthesis at sites where mRNAs are locally translated in response to stimuli.

**Methodology/Principal Findings:**

Here, we report the X-ray crystal structures of the non-canonical nuclear localization signals of the FXR1 and FXR2 autosomal paralogues of FMRP, which were determined at 2.50 and 1.92 Å, respectively. The nuclear localization signals of the FXR1 and FXR2 comprise tandem Tudor domain architectures, closely resembling that of UHRF1, which is proposed to bind methylated histone H3K9.

**Conclusions:**

The FMRP, FXR1 and FXR2 proteins comprise a small family of highly conserved proteins that appear to be important in translational regulation, particularly in neuronal cells. The crystal structures of the N-terminal tandem Tudor domains of FXR1 and FXR2 revealed a conserved architecture with that of FMRP. Biochemical analysis of the tandem Tudor doamins reveals their ability to preferentially recognize trimethylated peptides in a sequence-specific manner.

**Enhanced version:**

**This article can also be viewed as an enhanced version in which the text of the article is integrated with interactive 3D representations and animated transitions. Please note that a web plugin is required to access this enhanced functionality. Instructions for the installation and use of the web plugin are available in Text S1.**

## Introduction

Fragile X syndrome (FXS) is one of the most common inherited developmental disorders that is estimated to affect one in 4000 males and one in 8000 females[1]. Clinically, affected individuals face a broad range of intellectual and physical challenges, including IQ scores ranging between 20 and 70, mild abnormal facial features and macroorchidism in prepubescent males[2]. The underlying cause of FXS has been mapped to a large expansion of the CGG trinucleotide repeat in the 5′-untranslated region of the *FMR1*, *fragile X mental retardation 1* [3]. The typical repeat size in healthy individuals ranges from 7 to 40, but is expanded to more than 230 and exhibits abnormal hypermethylation in cases of the full mutation. Mutation in other areas of the *FMR1* gene have also been correlated with similar clinical presentation[4].

FMRP, Fragile X Mental Retardation Protein, and its autosomal paralogues, FXR1 and FXR2 (Fragile X mental Retardation Syndrome-related Protein 1 and 2, respectively), comprise a family of RNA-binding proteins[5], [6]. These proteins are highly similar to one another, exhibiting a sequence identity of greater than 60%, and also retain highly conserved domain architectures. The two ribonucleoprotein K homology domains (KH domains) and the cluster of arginine and glycine residues that constitute the RGG box, comprise a large region that is important for RNA binding and polyribosome association[7], [8]. FMRP has been shown to play an important role in translation control, both *in vivo* and *in vitro* [9], [10], [11]. Thus far, the wealth of data associated with FXS has pointed to a working model in which FMRP binds target mRNA in the nucleus to form a ribonucleoprotein complex which is transported to dendrites and spines. Here, FMRP is involved in the regulation of protein synthesis at sites where mRNAs are locally translated in response to stimuli[12]. FMRP has also been shown to interact with components of the miRNA pathway, including RISC proteins (reviewed in[13]).

While FMRP exhibits predominant cytoplasmic localization, data from a variety of sources have pointed to an ability of FMRP to shuttle into the nucleus[14], [15]. Immunogold staining has identified FMRP in the neuronal nucleoplasm and within nuclear pores^15^. In both *Xenopus tropicalis* and zebrafish embryos, FMRP is predominantly localized to the nucleus early in development[16], [17]. This altered localization has been correlated with periods in which no zygotic transcription is detectable, suggesting that the export of FMRP, and its paralogues, from the nucleus is dependent on mRNA synthesis[18]. More recently, the functional and physical association of FMRP with the primary RNA exporter Tab/NXF1, and with the active transcription units of the lampbrush chromosomes in amphibian oocytes, has provided the first direct lines of evidence of FMRP-mRNA associated within the nucleus[19].

The N-terminal region of FMRP, and the FXR1 and FXR2 paralogues, contains a nuclear localization signal between residues 1 and 184[8], [20], [21]. Prior structural studies of the FMRP protein identified a repeat of two domains within the NLS region that each closely resembles the Tudor domain of the SMN protein[22]. NMR-based titrations with ^15^N-labelled protein corresponding to the NLS region also suggested its ability to interaction with trimethylated substrates, though this was assessed only from binding assays with individual amino acids carrying single post-translational modifications[22]. In this work, we present the crystal structures of the NLS of the FXR1 and FXR2 paralogues and definitively classify this region as a member of the Royal Family comprising a tandem Tudor repeat. Extensive structural and biochemical analyses also suggest that this region recognizes tri-methylated protein substrates in a sequence-specific manner common to both paralogues.

## Results

### The NLS of FXR1 and FXR2 comprises a tandem Tudor domain

**Figure 1:**
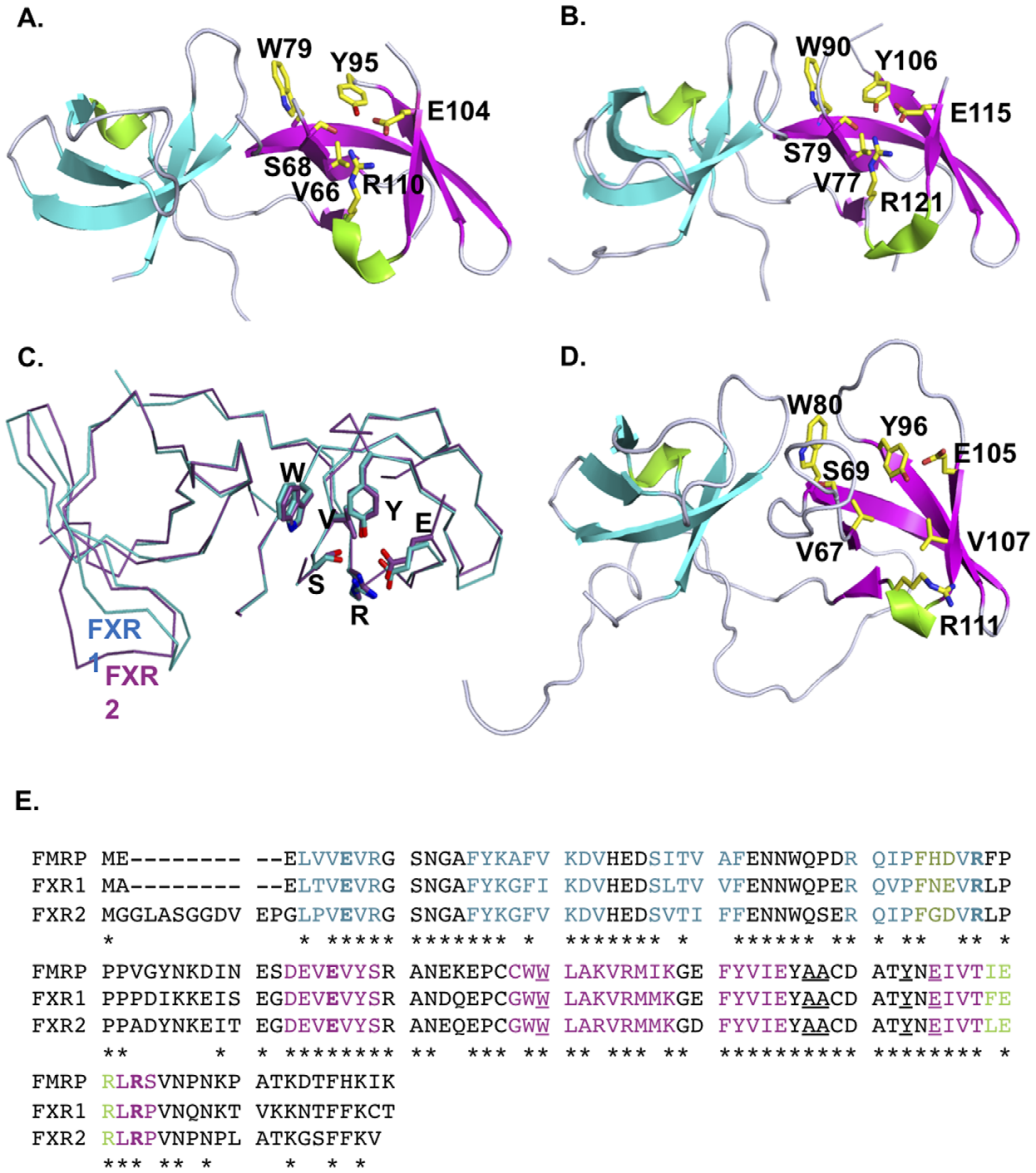
The crystal structures of FXR1 (A) and FXR2 (B) reveal a shared tandem Tudor domain architecture. Tud 1 domains are colored in cyan and Tud2 domains in magenta. Coiled regions are indicated in grey. The residues forming the aromatic cage of Tud2 are shown as in stick representation and are colored yellow. (C) FXR1 (cyan) and FXR2 (purple) align well and reveal a conserved interdomain orientation. (D The previously determined structure of FMRP (PDB 2BDK) also comprises the tandem Tudor architecture. The coloring is as described for the FXR1 and FXR2 panels. (E) The sequence alignment of the FXR proteins. Residues are colored in agreement with the β-strands of panels A, B, and D. Residues in bold correspond to the ionic lock, underlined residues exhibit alterations in the HSQC spectra on peptide titration, and the asterisks denote strictly conserved residues.

The crystal structures of residues 4 to 122 of the FXR1 (Figure 1A) and residues 13 to 136 of the FXR2 protein (Figure 1B) were determined at 2.5 and 1.92 Å, respectively (Table 1). Each structure was elucidated by the single anomalous dispersion (SAD) technique using a selenomethionine-substituted derivative. Superposition of the FXR1 and FXR2 tandem Tudor domains structures yields excellent alignment with a 1.0 Å r.m.s.d. (Figure 1C) and a sequence identity of 79% for the aligned regions (Figure 1E).

**Table 1:** Crystallographic and refinement statistics.

Data Collection		
	FXR1	FXR2
**Wavelength** (Å)	0.979	0.979
**Space Group**	R3	P2_1_2_1_2_1_
**Unit Cell Parameters** (Å)	a = 71.9b = 71.9	a = 34.6b = 54.7
	c = 94.1	c = 70.17
**Resolution** (Å)	50-2.50	100-1.92
	(2.59-2.50)	(1.96-1.92)^a^
**Reflections**		
Unique	6291 (643)	10551 (533)
Completeness (%)	99.9 (100)	99.0 (87.0)
Redundancy	4.0 (3.9)	6.1 (2.5)
**I/σ(I)**	11.3 (1.7)	24.2 (1.5)
**Rsym (I)** b	0.074 (0.547)	0.09 (0.96)

Refinement		
**Resolution** (Å)	35.93-2.50	43.11-1.92
**Reflections**		
Number	6000	10430 (589)
Completeness (%)	99.9 (99.6)	100 (80)
Test Set (%)	4.6	4.5
**R_work_**	21.8(21.0)	0.21 (0.26)
**R_free_**	26.1 (46.8)	0.25 (0.34)
**E.S.U.** (Å)^c^	0.205	0.103
**Contents of A.U.** d		
Protein Molecules/Atoms	1/805	1/873
Solvent	1	63
**Mean B-Factors** (Å^2^)		32.05
Protein	68.1	20.5
**Ramachandran Plot** (%)		
Preferred	95.7	99.0
Allowed	4.3	1.0
Outlier	0	0.0
**RMSD** e **from Target Geometry**		
Bond Lengths (Å)	0.017	0.021
Bond Angles (°)	1.760	1.729
**PDB ID**	3O8V	3H8Z

a Data for the highest resolution shell in parenthesis.

b R_sym_(I) = ∑_hkl_∑_i_| I_i_(hkl) - <I(hkl)>|/∑_hkl_ ∑_i_ | I_i_ (hkl)|; for n independent reflections and I observations of a reflection; I(hkl)>-average intensity of the I observations.

c E.S.U. – estimated overall coordinate error based on maximum likelihood.

d A.U. – asymmetric unit.

e RMSD – root mean squared deviation.

Alignment of the full tandem Tudor architecture with the N-terminal region of FMRP (Figure 1D, PDB 2BDK), which was previously determined by nuclear magnetic resonance experiments, reveals a highly conserved structural architecture with only 2 Å r.m.s. deviation when the tandem Tudors of either FXR1 or FXR2 are used for alignment. Minor deviations are observed only in the conformations of the highly flexible loops between all three paralogues, with little deviation observed in the assembly of the individual Tudor domains or the relative orientation of the N- and C-terminal Tudors (Figure 1).

**Figure 2:**
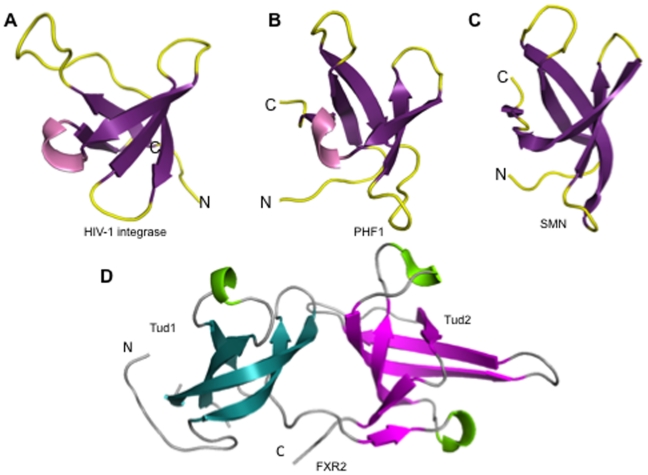
Structural similarity of the Fragile X Tudor domains with other β-barrel proteins. 3D Structures of: (A) the DNA binding domain of the HIV-1 integrase (PDB 1IHV); (B) the Tudor domain of the PHD finger protein 19 (PDB 2E5Q); (C) the Tudor domain of the human SMN protein (PDB 1G5V). These three structures are shown in the same orientation based on superposition. (D) Crystal structure of FXR2 is shown for comparison. The first Tudor (tud1) is colored in cyan and the second Tudor (tud2) is colored in purple.

The N-terminal Tudor domain (Tud1) spans residues 2 to 49 of FXR1 and residues 13 to 56 of the FXR2 protein. The Tud1 domain forms a canonical Tudor barrel that comprises five highly twisted antiparallel β-strands with a single 3_10_ helix residing between strands β4 and β5. Alignment of the Tud1 domains of FXR1 and FXR2 yields a 1.07 Å r.m.s deviation with 79% sequence identity. Structural comparison of the Tud1 domain with other proteins of known structure was performed with the Secondary Structure Matching server (SSM). The Tud1 domains from both FXR1 and FXR2 align well with the C-terminal DNA-binding domain of the HIV-1 integrase (Figure 2A, PDB 1qmc), for which the root mean square deviations are 1.49 and 1.3, respectively (Figure 2). Good structural alignments with r.m.s. deviations between 0.8 and 2 Å are also obtained for alignments with other Tudor domains, including those of PHF1 (PDB 2e5p), PHF19 (Figure 2B, PDB 2e5q), and the survival of motor neuron (SMN) protein (Figure 2C) PDB 1g5v).

The C-terminal Tudor (Tud2) domains of FXR1 and FXR2 yield a 1.08 Å r.m.s.d. on alignment with a 79% sequence identity for the aligned residues. These domains also show a high degree of structural homology with Royal Family member proteins, including the SMN protein (PDB 1g5v), PHF19 (2e5q), and the HIV-1 integrase binding domain (PDB 1ihv) (Figure 2). Good agreement was also observed on alignment with the malignant brain tumor repeat (MBT) domain of PHF20L1 (PDB 2jtf) and the metal response element-binding transcription factor 2 (PDB 2eqj). All alignments yielded r.m.s. deviations below 2 Å.

Alignment of the Tud1 with Tud2 for each of the paralogues also reveals a high degree of structural conservation between the individual Tudor domains (1.4 Å r.m.s.d.). However, while structurally the Tudors are very similar, the Tud1 domains exhibit much great sequence conservation with one another than is observed between the Tud 1 and Tud2 from a single protein. Sequence alignments of Tud1 with Tud2 result in 14% sequence and 17% identity between the Tudor domains of FXR1 and FXR2, respectively (data not shown).

### The tandem Tudor domain closely resembles that of UHRF1

The tandem arrangement of the FXR Tudor domains closely resembles that observed in the crystal structure of the E3 ubiquitin-protein ligase UHRF1 (PDB 3db4) (Figure 2D). The alignment of FXR1 with UHRF1 produces a 2.8 Å r.m.s.d, while that of FXR2 with UHRF1 results in a 2.5 Å r.m.s.d. The sequence identities are 12% and 11%, respectively, for the aligned regions.

### The Tudor domains utilize a highly charged interface for association

**Figure 3:**
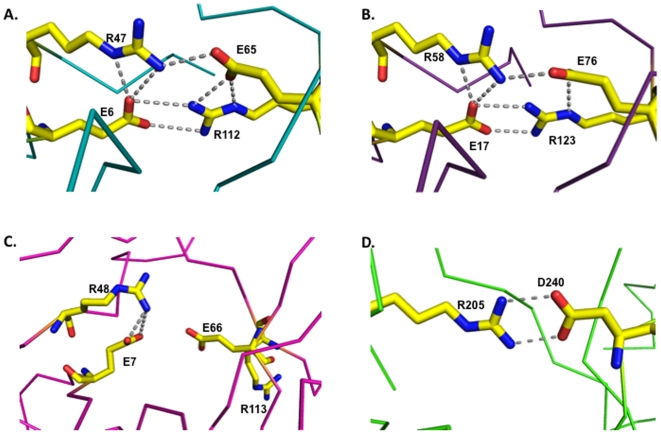
An interdomain ionic lock stabilizes the tandem Tudor architecture. The FXR1 (A) and FXR2 (B) domains are stabilized by extensive interactions between the charged residues at this interface. While the residues are conserved in the FMRP protein (C), the NMR structure suggested a slightly different domain orientation that results in a loss of salt bridging. (D) The UHRF1 interface is also stabilized by the formation of a salt bridge. The ribbon traces are colored to correspond with Figure 1 and residues comprising the ionic lock are colored in yellow for all panels.

While prior structural work also demonstrated the existence of two Royal Family domains within the nuclear localization signals of the FMRP paralogues, this crystallographic analysis has permitted the high resolution visualization of the interface between the individual Tudor domains for both FXR1 and FXR2 (Figure 3). The Tudor domains tilt toward one another to generate an interdomain angle of approximately 110°. Interaction between Tud1 and Tud2 creates an interface of 257.5 Å^2^ for FXR1 and 258.9 Å^2^ for FXR2. The orientation is stabilized in FXR1 by an ionic lock formed by inter-domain salt bridges between E6 and R47 from Tud1 and E65 and R112 from Tud 2 (Figure 3A). The corresponding residues for FXR2 are E17 and R58 from Tud1 and E76 and R123 from Tud2 (Figure 3B). Hydrogen bonds between the mainchain atoms of F14 from Tud1 (F25 in FXR2) and W78 (W89 in FXR2) of Tud2 lend further stability to this region. Finally, the C-terminal extension comprising residues V114 to K122 (or V125 to A131 in FXR2), which fold back along the base of the tandem Tudor domains toward the N-terminus, engages in extensive interactions with the intervening segment that tethers the individual Tudor domains. This results in the formation of several hydrogen bonds that assist in anchoring of the relative orientations of the domains. No ionic lock is visible in the NMR structure of the FMRP protein (Figure 3C), though the relevant residues are strictly conserved (Figure 1D), suggesting some degree of flexibility in the inter-domain orientation.

Strikingly, the relative orientation of the tandem Tudor domains of UHRF1 is highly similar to that of the Fragile X paralogues and is also stabilized by salt bridge formation between residues R205 and D240 (Figure 3D). Extensive interactions between the C-terminal tail of the UHRF1 tandem Tudor with the segment joining the individual Tudor domains is also retained, suggesting that these may be important features in the organization and structural stability for this arrangement of tandem Tudor domains.

### FXR1 and FXR2 recognize peptides in a sequence-specific and PTM-specific manner

**Figure 4:**
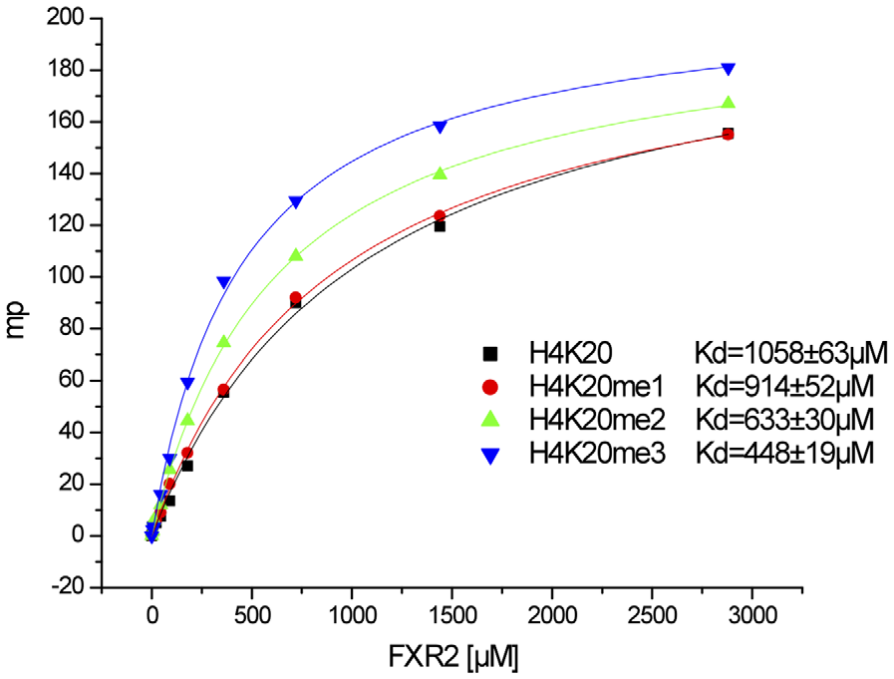
FXR1 and FXR2 preferentially recognizes trimethylated histone peptides. The fluorescence polarization binding curves for FXR2 and H4K20 peptides are shown as a example.

Royal family members have been widely studied for histone modification mark recognition[23], [24], [25], [26], [27]. The ability of the FXR1 and FXR2 paralogues to interact with peptides derived from histone tails carrying various post-translational modifications was assessed by fluorescence polarization assay. Titration of the tandem Tudor domains initially indicated potential interactions with H3K4me3, H3K9me3 and H4K20me3 (data not shown). Therefore, a systematic binding study was undertaken to assess the preferences of FXR1 and FXR2 for the varying methylation states at these lysine sites. The sequences of the peptides used in this study, along with their respective binding affinities are reported in Table 2. Both FXR1 and FXR2 proteins exhibit preference for trimethylated peptides over lower methylated lysine peptides of H4K20 (Figure 4 and Table 2). Reduced preference for trimethylation was also observed for interaction of the tandem Tudor domains of FXR1 and FXR2 with H3K4 or H3K9 peptides (data not shown). Furthermore, FXR2 shows some degree of preference for H4K20me3 peptide (Table 2). No detectable binding was detected for other H3- or H4-derived peptides (Table 2).

**Table 2:** Binding affinities of FXR1 or FXR2 Tudor domains to histone H3 or H4 methylated lysine peptides.

Peptides	Sequences	FXR1Kd (µM)	FXR2Kd (µM)
H4K20	G G A K R H R K V L R D N	>1 mM	1058±63
H4K20me1	G G A K R H R Kme1 V L R D N I Q		914±52
H4K20me2	G G A K R H R Kme2 V L R D		633±30
H4K20me3	G G A K R H R Kme3 V L R D	692±113	448±19
H3K4me3	A R T Kme3 Q T A R K S T	660±121	>1 mM
H3K9me3	A R T K Q T A R Kme3 S T G G K A	>1 mM	>1 mM
H3K27me3	Q L A T K A A R Kme3 S A P A	No binding	No binding
H3K36me3	P A T G G V Kme3 K P H R Y		No binding
H3K37me3	P A T G G V K Kme3 P H R Y		No binding
H3K79me3	E I A Q D F Kme3 T D L R Y		No binding

### NMR and molecular docking simulations suggest the Tud2 aromatic cage to be the site of ligand recognition

**Figure 5:**
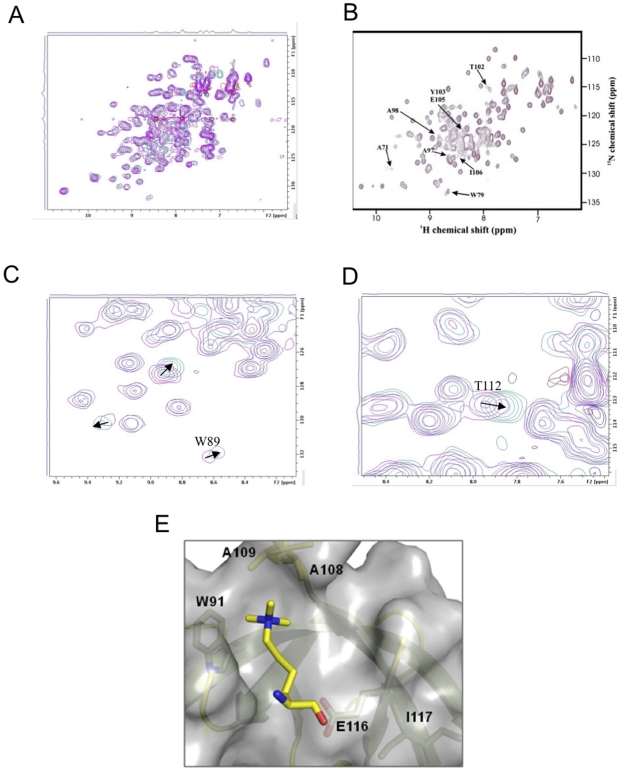
Recognition of trimethylated lysine by the Tud2 domain of FXR2. (A) Superposition of the HSQC spectra for the tandem Tudor domains of FXR2 in the presence (cyan) and absence (magenta) of the 1.5 molar excess H4K20me3 peptide. (B) HSQC spectra for FMRP reported in refenrence 22. (C) and (D) Specific chemical shifts corresponding to the predicted binding site for trimethylated lysine in FXR2-Tud2. (E) A model of trimethylated lysine recognition by FXR2-Tud2. Residues present in the crystal structure and that yield chemical shifts during titrations are indicated.

In agreement with the fluorescence polarization data, the addition of 1.5 molar excess of H4K20me3 peptide resulted in specific chemical shifts in the ^15^N-HSQC NMR spectrum of FXR2 (Figure 5A–D). The chemical shifts are quite similar to those reported for the titration of FMRP with trimethylated lysine[22]. Molecular docking simulations permitted the placement of trimethylated lysine in this site in a conformation consistent with the observed chemical shift (Figure 5E).

## Discussion

The FMRP, FXR1 and FXR2 proteins comprise a small family of highly conserved proteins that appear to be important in translational regulation, particularly in neuronal cells. The crystal structures of the N-terminal tandem Tudor domains of FXR1 and FXR2 were determined at 2.50 Å and 1.92 Å, respectively, and revealed a conserved architecture as compared with the same region of FMRP. Biochemical analysis of the FXR1 and FXR2 proteins suggested that both proteins preferentially recognize trimethylated histone peptides (Table 2).

The interaction of FMRP with trimethylated lysine residues was initially demonstrated via NMR titration, but not in the context of histone-derived peptides[22]. This interaction was of low affinity and required titration with 20-fold molar excess of ligand to induce specific amide resonance shifts for a pocket located in the second Tudor domain. The residues defining this pocket (Y95, W97, V66, and E104 for FXR1) are strictly conserved amongst the Fragile X paralogues. NMR titration experiments carried out for FXR2 resulted in similar shifting with only 1.5-fold molar excess of H4K20me3 peptide suggesting a specific interaction of higher affinity than for the methylated lysine alone.

Structural alignment of the tandem Tudors from the fragile X family revealed excellent conservation of the tandem Tudor assembly with that of the UHRF1 protein, for which crystallographic analysis has suggested an affinity for H3K9me3 (PDB 3db3). Despite the conservation of domain architecture, however, structural analysis of the interaction of H3K9me3 with the UHRF1 Tudor domains revealed Tud1 to be the module responsible for the recognition of this peptide. Analogous to the methyl-lysine binding site of the Fragile X paralogues, that of UHRF1 also comprises both aromatic (Phe, and Tyr) and polar (Asp and Asn) residues. For all these four proteins, each Tudor domain appears to possess pockets that may potentially recognize post-translation modifications on a single substrate, though multivalent interaction of tandem Tudor domains in this structural arrangement has yet to be demonstrated.

It has been previously demonstrated that FMRP becomes associated with its cargo mRNAs in the nucleus in a manner that is dependent on the presence of a non-classical nuclear localization signal (NLS)[19]. Removal of the NLS was also shown to compromise RNA binding, as well as association of the FMRP protein with its autosomal paralogue FXR1[19]. The ability of the Fragile X proteins to recognize methyl marks in a sequence-dependent fashion may suggest an involvement of methyllysine recognition in targeting these proteins within the nucleus, either to newly synthesized transcripts or in recognition of other components of FXR-mRNA complex that are also methylated. Clearly, elucidation of the *in vivo* function of the tandem Tudor domains of the FXR paralogues will be a critical component for modeling the roles of these proteins in mRNA trafficking and associated disease pathologies.

## Materials and Methods

### Cloning, expression and protein purification

DNA sequences corresponding to amino acids 2 to 132 of FXR1 and 14 to 137 of FXR2 were subcloned into the pET-28a-MHL vector via ligase-independent cloning. Recombinant His_6_-TEV-FXRs were expressed in a SGC-generated derivative strain of BL21 *Escherichia coli* with the pRARE plasmid for codon biased expression. Cells were grown in minimal media supplemented with selenomethionine (Molecular Dimensions Inc, Apopka, FL) in the presence of kanamycin and chloramphenicol at 37°C to an optical density of approximately 2.5. Protein expression was induced with 1 mM isopropyl-1-thio-D-galactopuranoside and the cell cultures continued for approximately 16 hours at 15°C. The cells were harvested via centrifugation and the resultant pellet stored at -80°C prior to purification.

The cell pellet from a 2 L culture was resuspended in 200 mL of lysis buffer consisting of PBS pH 7.2–7.5 (Bioshop, Burlington, ON), 250 mM NaCl, 5% glycerol, 10 µM β-mercaptoethanol, 1 mM PMSF, 5 µg mL^−1^ Benzonase, 0.2% CHAPS. The homogenized suspension was lysed via sonication and insoluble material removed via centrifugation. The clarified supernatant was passed over 5 mL of Ni-NTA resin (Qiagen Mississauga, ON) that had been pre-equilibrated with 20 mM Tris-Cl pH 8.0, 500 mM NaCl. The resin was washed with 50 column volumes of 20 mM Tris-Cl pH 8.0, 500 mM NaCl, 10 mM imidazole. Finally, the protein was eluted from the resin with 15 mL of 20 mM Tris-Cl pH 8.0, 250 mM NaCl, 250 mM imidazole, 5% glycerol. Further purity for each sample was achieved via Superdex 75 (GE Healthcare, Tyrone, PA) size exclusion chromatography in 20 mM Tris-Cl pH 7.5, 200 mM NaCl, 1 mM DTT.

The hexahistide purification tag was cleaved from FXR1 by the addition of 0.05 mg TEV protease per milligram of FXR1 protein, followed by incubation at 4°C for 12 hours. The sample was then passed over a Ni-NTA column and the flow-through collected for crystallization. The purification tag was not removed from FXR2.

### Crystallization

Crystals of diffraction quality were grown at 18°C using the sitting drop method for FXR1 and hanging drop method for FXR2 by mixing an equal volume of protein solution with reservoir solution. The proteins were concentrated to 10 mg mL^−1^ in 20 mM Tris-Cl, pH 7.0, 200 mM NaCl and 1 mM DTT prior to crystallization. The crystallization condition for FXR1 was 1.4 M (NH4)_2_SO_4_, 0.25 M NaCl, 0.1 M HEPES pH 6.8. FXR2 crystallized in 20–30% polyethylene glycol 3350, 0.2 M MgCl_2_, 0.1 M HEPES, pH 7.5. Crystals were harvested and soaked in the crystallization condition with 20–30% glycerol prior to freezing in liquid nitrogen.

### Structure determination of FXR1

Data for FXR1 were collected at the Canadian Macromolecular Crystallography Facility (CMCF) on beamline 08ID-1 at the Canadian Light Source (Saskatoon, SK, Canada) at 100K. Intensities were integrated and scaled using HKL2000[28]. The structure of FXR1 was determined using the single-wavelength anomalous dispersion (SAD) method utilizing the anomalous signal from selenium atoms. The positions of 2 selenium atoms were found with the program SHELXD[29] followed by heavy atom refinement and phasing using the maximum likelihood-based algorithm as implemented in the auto-SHARP program suite[30]. Phase improvement by density modification generated an interpretable experimental SAD map, allowing an initial model to be built using ARP/wARP[31]. Following alternate cycles of manual building using COOT[32] and restrained refinement using REFMAC[33], the presence of twinning was initially suspected owing to unusually elevated R/R_free_ residual factors (>30%) and later confirmed by intensity statistics calculations, as performed using the module phenix.xtriage[34]. Subsequent refinement in REFMAC[33] employing a merohedral twin model for space group R3 with twin operators (H K L) and (K H–L) immediately reduced R/R_free_ below 30%, revealing a minor twin fraction of 5.9%. The final model comprising one molecule of FXR1 refined to an R_work_ of 21.8% and R_free_ of 26.1%.

### Structure determination of FXR2

Single anomalous dispersion data were collected at the selenium K edge at 19-ID at the Advanced Photon Source (Chicago, IL, USA) at 100K. Data intensities were indexed and scaled with HKL2000[28]. Heavy atom substructure determination followed by phase refinement and density modification were carried out in SOLVE and RESOLVE[35], [36], respectively. Automated model building in ARP/wARP was utilized to generate the initial model. Manual model improvements and refinement were carried out in COOT[32] and REFMAC[33], respectively.

### Fluorescence polarization screening of peptide substrates

All peptides used for fluorescence polarization (FP) and isothermal titration calorimetry (ITC) measurements were synthesized by Tufts University Core Services (Boston, MA, USA). Fluorescence polarization assay was performed as reported previously[37], [38]. FXR1 and FXR2 were dialyzed into 20 mM Tris-Cl pH 8.0, 50 mM NaCl, 1 mM DTT and concentrated to 18.2 mg mL^−1^ (1.03 mM) and 39.2 mg mL^−1^ (2.4 mM) respectively. Serial twofold dilutions were then made to produce 25 µl of ½, ¼, 1/8, 1/16, 1/32, 1/64, 1/128, 1/256, 1/512, 1/1024 diluted samples. Buffer without protein was used as blank control. The fluoresceinated peptides H3K4Me3, H3K9Me3, H3K27Me3, H4K20Me3 and H4K20Me0 were each added to above samples to a final concentration of 100 nM. Samples were incubated at room temperature for 30 minutes and centrifuged prior to use. The anisotropy of the sample was recorded with a Synergy 2 Multi-Mode microplate Reader (BioTek, Winooski, VT)). The reported polarization value was fit and plotted by Origin 6.1 (OriginLab, Northampton, MA).

### NMR titrations of histone derived peptides

Preparation of isotopically labeled FXR2 Tudor domain followed similar purification steps as above except that instead of LB media, M9 media containing 1 g/L ^15^N NH_4_Cl, 4 g L^−1^ [^12^C_6_]-D glucose was used. To investigate the binding of FXR2 Tudor domain to methylatd histone peptides, ^15^N-^1^H HSQC spectra were collected with ^15^N-labeled FXR2 samples, free and with additions of increasing amounts of unlabeled histone peptides. All NMR experiments were performed at 298K with Bruker Avance 500 MHz spectrometers equipped. ^15^N-FXR2 was concentrated to 0.3 mM, prepared in 20 mM NaH_2_PO_4_, pH 6.7, 50 mM NaCl, 1 mM EDTA, 1 mM DTT, and 93% H_2_O/7% D_2_O.

#### Docking of trimethylated lysine in the FXR2 aromatic cage

Docking simulations using trimethylated-lysine were carried out in AutoDock 4.2 with AutoDockTool-1.5.4. Static coordinates for the ligand binding site residues were employed and the docking carried out using the genetic algorithm. Default docking parameters were utilized. Clusters were scored and the lowest energy conformational clusters analyzed.

## Supporting Information

Text S1 Instructions for installation and use of the required web plugin (to access the online enhanced version of this article).
(PDF)

Datapack S1 Standalone iSee datapack - contains the enhanced version of this article for use offline. This file can be opened using free software available for download at http://www.molsoft.com/icm_browser.html.
(ICB)
